# Predicting Accumulation of Intermediate Compounds in Nitrification and Autotrophic Denitrification Processes: A Chemical Approach

**DOI:** 10.1155/2019/2051986

**Published:** 2019-07-07

**Authors:** José Luis Campos, Jacques Dumais, Juan Pablo Pavissich, Oscar Franchi, Dafne Crutchik, Marisol Belmonte, Martín Faúndez, Lorena Jorquera, Alba Pedrouso, Anuska Mosquera-Corral, Ángeles Val del Río

**Affiliations:** ^1^Facultad de Ingeniería y Ciencias, Universidad Adolfo Ibáñez, Avda. Padre Hurtado 750, Viña del Mar, Chile; ^2^Center of Applied Ecology and Sustainability (CAPES), Santiago, Chile; ^3^Department of Environment, Faculty of Engineering, University of Playa Ancha, Avenida Leopoldo Carvallo 270, 2340000 Valparaíso, Chile; ^4^Chemical and Environmental Engineering Department, Technical University Federico Santa María, Ave. España 1680, Valparaíso, Chile; ^5^Escuela de Ingeniería en Construcción, Facultad de Ingeniería, Pontificia Universidad Católica de Valparaíso, Avenida Brasil 2147, Valparaíso, Chile; ^6^Department of Chemical Engineering, Institute of Technology, Universidade de Santiago de Compostela, E-15705 Santiago de Compostela, Spain

## Abstract

Nitrification and sulfur-based autotrophic denitrification processes can be used to remove ammonia from wastewater in an economical way. However, under certain operational conditions, these processes accumulate intermediate compounds, such as elemental sulphur, nitrite, and nitrous oxide, that are noxious for the environment. In order to predict the generation of these compounds, an analysis based on the Gibbs free energy of the possible reactions and on the oxidative capacity of the bulk liquid was done on case study systems. Results indicate that the Gibbs free energy is not a useful parameter to predict the generation of intermediate products in nitrification and autotrophic denitrification processes. Nevertheless, we show that the specific productions of nitrous oxide during nitrification, and of elemental sulphur and nitrite during autotrophic denitrification, are well related to the oxidative capacity of the bulk liquid.

## 1. Introduction

Removal of reduced nitrogen species from wastewater is conventionally carried out by means of nitrification and denitrification biological processes, where ammonia nitrogen (NH_4_^+^) is first converted to nitrate (NO_3_^−^) and then to nitrogen gas (N_2_) [1]. Wastewater nitrification occurs under aerobic conditions whereas denitrification is anaerobic. Nitrification is conducted in two consecutive steps by nitrifying microorganisms: ammonia conversion into nitrite by ammonia oxidizers (i.e., partial nitrification) and then nitrite conversion into nitrate by nitrite oxidizers. The most studied nitrifiers are ammonia-oxidizing bacteria (AOB), such as* Nitrosomonas*, and nitrite-oxidizing bacteria (NOB), where* Nitrobacter* is the most referenced genus. Nitrifiers are chemolithotrophic and their activity decreases as the pH is reduced below neutrality [2].

Typically, denitrification relies on the oxidation of organic carbon by heterotrophic bacteria, and readily biodegradable carbon sources such as methanol, ethanol, and acetate, must be added externally to treatment plants [3]. Organic electron donors are expensive and have high biomass yields, leading to higher operational costs and sludge production [4]. Autotrophic denitrification is an alternative process for the reduction of nitrate or nitrite (NO_2_^−^), which is accomplished by oxidation of inorganic electron donors, including different forms of sulfur such as sulphide [5]. Among denitrifying bacteria that are able to use sulfur compounds as electron donors is* Thiobacillus denitrificans* which is the most studied. This species of bacteria has its optimal growth conditions at pH 7.5-8.0 [6]. Autotrophic denitrification supported by sulfur poses several advantages over common denitrification, because sulfur compounds are more cost-effective and have much lower sludge production [7]. Thus, the combination of nitrification and sulfur-based denitrification is attracting increasing interest in recent years. Particularly, these processes are gaining attention in order to remove nitrogen from anaerobic reactor effluents containing low organic matter and high ammonia and sulphide (S^2-^) concentrations [7]. However, under certain operational conditions ammonia and sulphide removal processes generate undesirable intermediates such as nitrous oxide (N_2_O), nitrite, and elemental sulphur (S^0^). Nitrous oxide is a potent greenhouse gas, which can be produced in the aerobic nitrification process by AOB in presence of low dissolved oxygen (DO) concentrations and nitrite accumulation [8]. Under aerobic conditions, AOB metabolism can generate N_2_O through hydroxylamine oxidation or nitrite oxidation [8] while NOB are not able to produce this compound in presence of oxygen [9]. On the other hand, the accumulation of nitrite and elemental sulphur can occur during autotrophic denitrification [10]. The presence of these compounds should be avoided to maintain the stability of the process due to the toxic effect of nitrite on the sulphide oxidation rate [11] and the decrease of biomass activity due to the formation of sulphur precipitates [12]. The accumulation of both compounds could be related to the S/N ratio of the influent [13, 14]. S/N ratio may have a major influence on the distribution of sulphide oxidation and nitrate reduction end-products and on their simultaneous removal [10, 15, 16].

Under a thermodynamic point of view, the formation of redox intermediates could be predicted considering the Gibbs free energy (∆G) of the potential reactions, normalized to the number of moles of electrons (e^−^) transferred in such reactions, since the most energetically favorable reactions would be preferentially used by microorganisms [18]. Alternatively, to predict the reactions that can take place in a specific aqueous system, Scott and Morgan [17] proposed the use of a conservative parameter called oxidative capacity (OXC) which represents the total number of transferable electrons in a given system. This parameter is defined as the equivalent sum of all oxidants that can be reduced with a strong reductant to an equivalence point. At every equivalence point a particular electron condition defines a reference level of electrons. By using the OXC concept, the information about the chemical composition of the bulk liquid is condensed into a single descriptive parameter which can be easily calculated as

(1) $$\begin{array}{c} \text{OXC }\left(\;equivalents/L\;\right)=\Sigma n_{i}\cdot {\left[\;Ox\;\right]}_{i}-\Sigma n_{i}\cdot {\left[\;Red\;\right]}_{i} \end{array}$$

where [Ox]_i_ and [Red]_i_ represent the concentration (molar) of the individual oxidants and reductants of the system and n_i_ is the number of equivalent electrons that are transferred.

The objective of this research is to determine if the Gibbs free energy or the oxidative capacity are useful parameters to predict the possible accumulation of intermediate compounds during nitrification and autotrophic denitrification processes.

## 2. Materials and Methods

### 2.1. Experimental Data

The production of intermediate compounds in nitrification and autotrophic denitrification processes was analyzed using data from experimental bench-scale reactors. Measurements of N_2_O emissions in nitrification were those obtained by Campos et al. [9], during the operation of a nitrifying biofilm airlift suspended (BAS) reactor under different dissolved oxygen concentrations. The nitrifying BAS reactor was of 2.6 L, continuously fed with a synthetic medium containing 500 mg NH_4_^+^-N per liter, operated at 23°C and a hydraulic retention time of 8 h under three different DO concentration conditions: 1, 2, and 5 mg O_2_ per liter. After 1 week of operation, once a constant composition of the liquid phase was achieved for each condition, quick changes (3-5 min in length) in the DO concentration were carried out (Table 1). DO concentrations tested in these quick-change assays were 0.5, 1, 2, and 5 mg O_2_ per liter. Thus, a total of 12 conditions were evaluated for the production of N_2_O (Table 1). Periodical samples of the outlet gas were taken until verifying that a constant concentration of N_2_O was reached for each DO concentration tested.

The accumulation of elemental sulfur and nitrite during autotrophic denitrification was studied using data from Fajardo et al. [12], for a denitrifying sequencing batch reactor (SBR) simultaneously removing nitrate and sulphide operated during 220 days. The SBR had a working volume of 1 L and was fed with a synthetic medium containing nitrate and sulphide (500 mg NO_3_^−^-N/L and 100-450 mg S^−2^-S/L) using different loading rates in 9 stages (Table 1). The reactor was operated at 30°C and at a fixed hydraulic retention time of 1 d.

### 2.2. Calculations

The reactions involved during the analyzed processes of ammonia oxidation and of autotrophic denitrification with sulphur compounds and related to the production of NO_2_^−^, N_2_O, and S^0^ as intermediate compounds, are listed in Table 2. The Gibbs free energy value was calculated taking into account the concentrations of the different compounds for each operational condition, normalized to the number of moles of e^−^ transferred in the reaction (∆G/e^−^).

**Figure 1:**
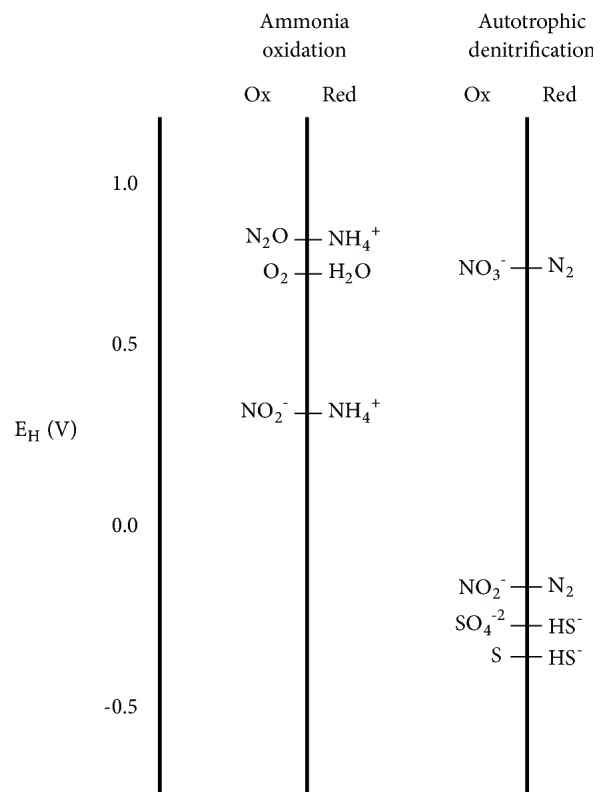
“Redox ladder” for the ammonia oxidation and autotrophic denitrification processes (adapted from Scott and Morgan [17]).

To determine the oxidative capacity for the different operating conditions, first, in order to make a “redox ladder” [17], the redox potential of each half reaction involved in ammonia oxidation and autotrophic denitrification was calculated as a function of pH (Table 3), using the experimental data summarized in Table 1.  Table 4 shows that, for both nitrification and denitrification, the related redox potential values were similar for all the operational stages. A “redox ladder” was set using the calculated potentials (Figure 1). In the case of ammonia oxidation, NH_4_^+^ was selected as the electron reference level from the redox ladder while bisulphide (HS^−^) was chosen for autotrophic denitrification, since these are the main species at the analyzed pH conditions. Then, the OXC was calculated according to (2) and (3) for ammonia oxidation and denitrification processes, respectively:

(2) OXC equivalents/L=4·DO/16000+6·NO2−-N/14000

(3) OXC equivalents/L=5·NO3−-N/14000+3·NO2−-N/14000+8·SO4−2-S/32000

where [DO] is the dissolved oxygen concentration (mg O_2_/L), [NO_2_^−^-N] is the nitrogen concentration as nitrite (mg NO_2_^−^-N/L), [NO_3_^−^-N] is the nitrogen concentration as nitrate (mg NO_3_^−^-N/L), and [SO_4_^−2^-S] is the sulphur concentration as sulphate (mg SO_4_^−2^-S/L).

In order to obtain a specific rate of production intermediate compounds for each operational condition analyzed, the production of NO_2_^−^, N_2_O, and S^0^ was normalized using the measured biomass as volatile suspended solids (VSS) [9, 12].

### 2.3. Statistical Analyses

Simple linear regression analysis between the calculated OXC (explanatory variable) and the measured production of intermediate species (dependent variable) was performed using XLSTAT® software (Addinsoft, France). In order to evaluate the fitting of the regression models, regression characteristic (*p*-values and R^2^) and standardized residuals were studied. The confidence and prediction intervals were calculated by using the* F* distribution and analysis of variance (ANOVA) test.* P*‐value ≤ 0.05 was considered significant. A 95% prediction interval was determined, being the range in which one can expect any individual value to fall into 95% of the time.

## 3. Results and Discussion

### 3.1. Gibbs Free Energy

**Figure 2:**
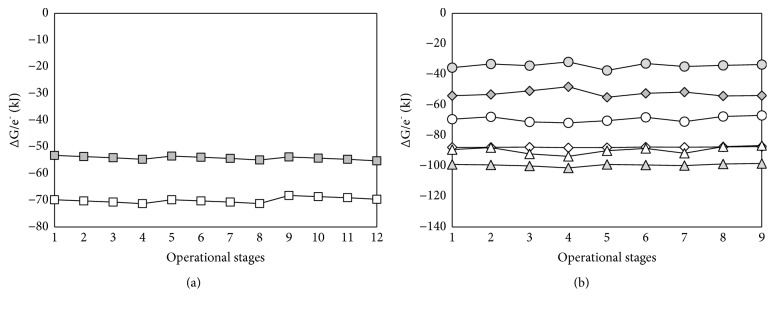
Gibbs free energy (kJ) per electron mol transferred under the conditions of the different operational stages: (a) ammonia oxidation: ■ reaction (i); □ reaction (ii) and (b) autotrophic denitrification: ◆ reaction (iii); ◊ reaction (iv); *∙* reaction (v); *ο* reaction (vi); ▲ reaction (vii); ∆ reaction (viii)). All reactions are described in Table 2.

The calculation of the Gibbs free energy shows that, for all of the operational conditions tested in the nitrification experiments, oxidation of ammonia to nitrous oxide provides higher energy per mol of e^−^ transferred to microorganisms than its oxidation to nitrite (Figure 2(a)). This fact could justify that nitrous oxide production was always detected although the predominant product was always nitrite (Table 1). The literature reports that N_2_O could be also generated during heterotrophic denitrification [9]. However, in this process, the energy available in the NO_3_^−^/NO_2_^−^ reduction to N_2_ was higher compared with the reduction to N_2_O [18] and, therefore, the Gibbs free energy does not explain nitrous oxide generation. The generation of N_2_O during heterotrophic denitrification is generally associated to the effect of environmental conditions on the different nitrogen oxide reductases involved in the four reductive steps of complete denitrification. Denitrification enzymes receive their electrons from a common source (i.e., the ubiquinone/ubiquinol pool of the respiratory electron transport chain [19]), and limitations in the electron supply rate or in substrate availability can result in electron competition among these enzymes and accumulation of nitrogen oxide intermediates [20]. Also, it has been shown that pH affects the electron donor oxidation rate supplying electrons and the nitrogen reductases activity [21]. In the case of ammonia oxidation (such as the observed in the studied experiments), it has been shown that the production of N_2_O can be related to an imbalanced metabolic activity and enzymatic regulation of AOB, especially under cyclic transitions in DO concentrations, or to chemical decomposition and oxidation of intermediate compounds [8]. Therefore, the key factors controlling intermediate compounds formation such as N_2_O during nitrification may not only be energy availability but also environmental conditions.

For the autotrophic denitrification experiments, according to the calculated Gibbs free energy (Figure 2(b)), the most thermodynamically favorable reactions are those where elemental sulfur is consumed (reactions (vii) and (viii) of Table 2), which would explain the consumption of this compound observed during the operational stages 6, 8, and 9 (Table 1). Nevertheless, according to the free energy calculations the least favorable reactions are those where elemental sulfur is the end product (reaction (iii) and (v) of Table 2). This fact that does not agree with the experimental results found during stages 2, 3, 4, and 7 where S^0^ accumulation was observed. On the other hand, despite the fact that the reduction of nitrate into nitrogen gas provides more energy than its reduction to nitrite (Figure 2(b)), NO_2_^−^ was detected during almost all operational stages of autotrophic denitrification process (Table 1). In sulfur-based autotrophic denitrification systems the formation of intermediate compounds has been related to the feeding S/N ratio and the operational conditions [9, 16], which are not evaluated using free energy analyses.

According to Seto and Iwasa [22], the behavior of chemotrophic microorganisms under anaerobic conditions is affected by the low level of energy available from redox reactions and, therefore, it would be expected that Gibbs free energy per electron mol transferred (∆G/e^−^) was an appropriate parameter to predict the accumulation of intermediate products at least for autotrophic denitrification. Nevertheless, the discrepancies between experimental data and those obtained by the theoretical calculations indicate the opposite.

### 3.2. Oxidative Capacity

**Figure 3:**
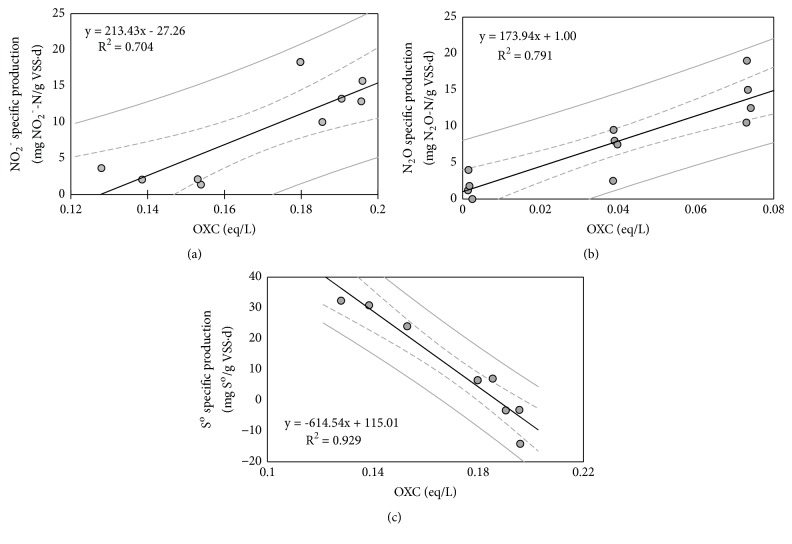
Regression lines between the specific production of nitrous oxide (a) and elemental sulphur (b) and nitrite (c) for each operational stage and the oxidative capacity of the bulk liquid. Dashed grey lines represent the 95% confidence interval, and solid grey lines represent the 95% prediction interval (in the case of elemental sulphur, negative production values indicate an overall consumption of the S^o^ accumulated in the system).

Analyses by linear regression showed significance and a strong to very strong relationship between the OXC of the bulk liquid and the production of intermediate compounds, in nitrification and autotrophic denitrification processes (Figure 3). In the case of nitrification, N_2_O specific production increases with the increase of the OXC of the bulk liquid (Figure 3(a), R^2^ = 0.704,* p* < 0.05). That is, N_2_O formation is promoted by both high DO and nitrite concentration. This agrees with the results of He et al. [23] who observed that N_2_O production was favored by high redox potential inside nitrifying biofilms. However, some studies reported that N_2_O formation decreased when the system was operated at high DO levels [24]. This cannot be attributed to the own effect of the dissolved oxygen but to the decrease of nitrite accumulation during the operation at high DO concentrations. In fact, Castro-Barros et al. [25] found an increase in the production of nitrous oxide at higher DO concentrations when nitrite was added to a nitrifying system. Moreover, high oxidative capacity and redox potential values are already reported in the literature as the most important parameters responsible for N_2_O production during nitrification in soils [4].

In the case of autotrophic denitrification, sulphur generation seems to be promoted by less oxidative environments while the opposite trend is observed for nitrite (Figures 3(b) and 3(c), R^2^ = 0.791 and 0.929,* p* < 0.05). Generally, the formation of both intermediate compounds is related to the feeding S/N ratio: an excess of electron donor causes the accumulation of elemental sulphur while an excess of electron acceptor leads to the generation of nitrite [13, 14]. Nevertheless, this is not a valid criterion to predict the formation of intermediates since some studies showed that S^o^ can be accumulated even under sulphide limitation conditions [10, 12] and, on the other hand, nitrite production was observed in the systems operated with an excess of sulphide [26]. Accumulation of S^o^ during autotrophic denitrification is also related to high pH values (> 8.5) [12]. This could be attributed to the effect of the pH on the redox state, probably due to a shift in the H_2_S/HS^−^/S^2-^ equilibrium, since higher pH values lead to lower redox potentials (lower oxidative capacity). This agrees with the results obtained in sulfide-oxidizing bioreactors showing that the formation of elemental sulfur is optimal at low redox potentials [27]. A similar tendency has also been observed during the aerobic oxidation of sulphide: the yield of S^o^ increases as the DO concentration decreases due to the decrease of the redox potential [28].

The redox potential corresponds to the activity of the electrons present in the bulk liquid that influences the NAD^+^/NADH ratio within cells. This ratio controls gene expression and enzyme synthesis for the overall cell metabolic activities [29]. Therefore, it is reasonable to think that the redox potential value inside bioreactors can affect the metabolite generation and, therefore, the spectrum of products obtained depending on the extracellular redox conditions. In fact, electrofermentation is a novel technique that is being used to change the overall performance in mixed-culture fermentations, by altering both microbial community structure and metabolic patterns [30]. Also, electrochemical control of the redox potential in mixed culture bioreactors has been shown to regulate microbial metabolites production [31].

In summary, the results show that there is good agreement between calculated OXC values and the specific rate of production of nitrous oxide, elemental sulfur, and nitrite, as intermediate compounds in the analyzed nitrification and autotrophic denitrification systems. This suggests that the OXC calculation can be used to assess and predict the generation of these intermediate compounds.

## 4. Conclusions

The value of the Gibbs free energy calculated for the evaluated operational conditions cannot be used in order to predict the formation of nitrous oxide, elemental sulphur, and nitrite during nitrification and autotrophic denitrification processes. Nevertheless, the oxidative capacity of the bulk liquid appears as a useful tool to predict the accumulation of these intermediates. The oxidative capacity is a parameter simple to calculate and may provide a valuable starting point for the evaluation of the accumulation of undesirable intermediate compounds in wastewater treatment systems.

## Figures and Tables

**Table 1:** Summary of performed experiments of nitrification and autotrophic denitrification processes [9, 12] for the evaluation of the production of N_2_O, S^0^ and NO_2_^−^ as intermediate compounds (subscript “i” indicates “influent” and subscript “e” indicates “effluent”).

Nitrification	pH	Temperature	NH_4_^+^-N_i_	NH_4_^+^-N_e_	NO_2_^−^-N_e_	NO_3_^−^-N_e_	DO tested		N_2_O-N production	
		(°C)	(mg/L)	(mg/L)	(mg/L)	(mg/L)	(mg/L)		(g N_2_O-N/g VSS·d)	
Stage 1	7.5	23	500	124	170	170	0.5		10.6	
Stage 2	7.5	23	500	124	170	170	1.0		18.7	
Stage 3	7.5	23	500	124	170	170	2.0		13.8	
Stage 4	7.5	23	500	124	170	170	5.0		12.4	
Stage 5	7.5	23	500	122	90	236	0.5		2.3	
Stage 6	7.5	23	500	122	90	236	1.0		9.8	
Stage 7	7.5	23	500	122	90	236	2.0		7.5	
Stage 8	7.5	23	500	122	90	236	5.0		6.6	
Stage 9	7.5	23	500	9	3	451	0.5		1.2	
Stage 10	7.5	23	500	9	3	451	1.0		4.0	
Stage 11	7.5	23	500	9	3	451	2.0		1.8	
Stage 12	7.5	23	500	9	3	451	5.0		0.0	

Autotrophic denitrification	pH	Temperature	NO_3_^−^-N_i_	S^2−^-S_i_	S^2−^-S_e_	NO_2_^−^-N_e_	NO_3_^−^-N_e_	SO_4_^−2^-S_e_	S^0^ specific production	NO_2_^−^ specific production
		(°C)	(mg/L)	(mg/L)	(mg/L)	(mg/L)	(mg/L)	(mg/L)	(mg S^0^/g VSS·d)	(mg NO_2_^−^-N/g VSS·d)

Stage 1*∗*	7.8	30	450	200	-* *-	-* *-	-* *-	-* *-	-* *-	-* *-
Stage 2	8.0	30	450	250	0.9	110	294	205	6.5	18.3
Stage 3	8.6	30	450	300	0.7	12	291	128	30.8	2.1
Stage 4	9.3	30	450	200	1.0	22	362	24	32.3	3.6
Stage 5*∗*	7.5	30	450	100	-* *-	-* *-	-* *-	-* *-	-* *-	-* *-
Stage 6	8.2	30	450	150	0.9	94	306	266	-13.0	15.7
Stage 7	8.4	30	450	300	1.0	13	309	160	24.0	2.1
Stage 8	7.7	30	450	350	0.9	77	239	375	-3.2	12.9
Stage 9	7.7	30	450	450	0.9	79	154	474	-3.3	13.2

**∗**Stable values were not achieved

**Table 2:** Reactions involved during ammonia oxidation and autotrophic denitrification processes, number of e^−^ transferred and ∆G (kJ) for each reaction.

Process		Reaction	e^−^	∆G (kJ)*∗*
Partial nitrification				

	(i)	NH_4_^+^ + 3/2 O_2_ → NO_2_^−^ + 2 H^+^ + H_2_O	6	-273.0+RTln⁡NO2-∙[H+]2[NH4+]∙[O2]3/2
	(ii)	2 NH_4_^+^ + 2 O_2_ → N_2_O + 2 H^+^ + 3 H_2_O	8	${-526.6+RT\ln\frac{\left[N_{2}O\right]∙[H^{+}{]}^{2}}{{\left[NH_{4}^{+}\right]}^{2}∙{\left[O_{2}\right]}^{2}}}_{\;\;}$

Autotrophic denitrification				

	(iii)	5 HS^−^ + 2 NO_3_^−^ + 7 H^+^ → 5 S + N_2_ + 6 H_2_O	10	-990.1+RTln⁡[N2][HS-]5∙[NO3-]2∙[H+]7
	(iv)	5 HS^−^ + 8 NO_3_^−^ + 3 H^+^ → 5 SO_4_^−2^ + 4 N_2_ + 4 H_2_O	40	$-3726.9+RT\ln\frac{{\left[{SO}_{4}^{-2}\right]}^{5}∙{\left[N_{2}\right]}^{4}}{{\left[{HS}^{-}\right]}^{5}∙{\left[{NO}_{3}^{-}\right]}^{8}∙{\left[H^{+}\right]}^{3}}$
	(v)	HS^−^ + NO_3_^−^ + H^+^→ S + NO_2_^−^ + H_2_O	2	$-136.6+RT\ln\frac{\left[{NO}_{2}^{-}\right]}{\left[{HS}^{-}\right]∙\left[{NO}_{3}^{-}\right]∙[H^{+}]}$
	(vi)	HS^−^ + 4 NO_3_^−^ → SO_4_^−2^ + H^+^ + 4 NO_2_^−^	8	$-499.7+RT\ln\frac{\left[{SO}_{4}^{-2}\right]∙[H^{+}]{∙\left[{NO}_{2}^{-}\right]}^{4}}{[{HS}^{-}]∙{\left[{NO}_{3}^{-}\right]}^{4}}$
	(vii)	5 S + 6 NO_3_^−^ + 2 H_2_O → 5 SO_4_^−2^ + 4 H^+^ + 3 N_2_	30	-2736.8+RTln⁡SO4-25∙[H+]4∙N23NO3-6
	(viii)	S + 3 NO_3_^−^ + H_2_O → SO_4_^−2^ + 2 H^+^ + 3 NO_2_^−^	6	${-414.9+RT\ln\frac{\left[{SO}_{4}^{-2}\right]∙{\left[H^{+}\right]}^{2}{∙\left[{NO}_{2}^{-}\right]}^{3}}{{\left[{NO}_{3}^{-}\right]}^{3}}}_{\;\;}$

*∗*R is the ideal gas constant (8.31·10^−3^ kJ/mol·K); T is the operational temperature (K); concentrations are given as molar (M).

**Table 3:** Half reactions involved during ammonia oxidation and autotrophic denitrification written as reduction processes (E_H_ was calculated considering [Ox]/[Red]=1 [15]).

Process	Half reaction	E_H_ (V)*∗*
Partial nitrification		

	1/4 O_2_ + H^+^+ e^−^ → 1/2 H_2_O	$1.23-\frac{2.303∙RT}{F}∙pH$
	1/8 N_2_O + 5/4 H^+^+ e^−^ → 1/8 H_2_O + 1/4 NH_4_^+^	$2.04-\frac{2.303∙RT}{F}∙\frac{5}{4}∙pH$
	1/6 NO_2_^−^ + 4/3 H^+^ + e^−^ → 1/6 NH_4_^+^ + 1/3 H_2_O	0.89-2.303∙RTF∙43∙pH

Autotrophic denitrification		

	1/5 NO_3_^−^ + 6/5 H^+^ + e^−^ → 1/10 N_2_ + 3/5 H_2_O	$1.25-\frac{2.303∙RT}{F}∙\frac{6}{5}∙pH$
	1/3 NO_2_^−^ + 4/3 H^+^ + e^−^ → 1/6 N_2_ + 2/3 H_2_O	$0.42-\frac{2.303∙RT}{F}∙\frac{4}{3}∙pH$
	1/8 SO_4_^−2^ + 9/8 H^+^ + e^−^ → 1/8 HS^−^ + 1/2 H_2_O	$0.25-\frac{2.303∙RT}{F}∙\frac{9}{8}∙pH$
	1/2 S + 1/2 H^+^ + e^−^ → 1/2 HS^−^	-0.11-2.303∙RTF∙12∙pH

*∗*F is the Faraday constant (96,500 C/mol)

**Table 4:** E_H_ calculation of the redox half-reactions involved during ammonia oxidation and autotrophic denitrification processes under the operating conditions of each studied stage.

Nitrification	N_2_O reduction to NH_4_^+^	O_2_ reduction to H_2_O	NO_2_^−^ reduction to NH_4_^+^	
	(V)	(V)	(V)	
Stage 1	1.49	0.79	0.30	
Stage 2	1.49	0.79	0.30	
Stage 3	1.49	0.79	0.30	
Stage 4	1.49	0.79	0.30	
Stage 5	1.49	0.79	0.30	
Stage 6	1.49	0.79	0.30	
Stage 7	1.49	0.79	0.30	
Stage 8	1.49	0.79	0.30	
Stage 9	1.49	0.79	0.30	
Stage 10	1.49	0.79	0.30	
Stage 11	1.49	0.79	0.30	
Stage 12	1.49	0.79	0.30	

Autotrophic denitrification	NO_3_^−^ reduction to N_2_	NO_2_^−^ reduction to N_2_	SO_4_^−2^ reduction to HS^−^	S reduction to HS^−^
	(V)	(V)	(V)	(V)

Stage 1	0.70	-0.19	-0.27	-0.34
Stage 2	0.68	-0.21	-0.28	-0.35
Stage 3	0.64	-0.26	-0.32	-0.36
Stage 4	0.59	-0.31	-0.37	-0.38
Stage 5	0.72	-0.17	-0.25	-0.33
Stage 6	0.67	-0.23	-0.29	-0.35
Stage 7	0.66	-0.24	-0.31	-0.36
Stage 8	0.70	-0.19	-0.26	-0.34
Stage 9	0.70	-0.19	-0.26	-0.34

## Data Availability

The data used to support the findings of this study are included within the article.
